# Human Genetic Ancestral Composition Correlates with the Origin of *Mycobacterium leprae* Strains in a Leprosy Endemic Population

**DOI:** 10.1371/journal.pntd.0004045

**Published:** 2015-09-11

**Authors:** Nora Cardona-Castro, Edwin Cortés, Camilo Beltrán, Marcela Romero, Jaime E. Badel-Mogollón, Gabriel Bedoya

**Affiliations:** 1 Instituto Colombiano de Medicina Tropical—Universidad CES, Sabaneta, Antioquia, Colombia; 2 Grupo GENMOL, Instituto de Biología Universidad de Antioquia, Medellín, Colombia; 3 Skandha EIT S.A.S., Medellin, Colombia; Fondation Raoul Follereau, FRANCE

## Abstract

Recent reports have suggested that leprosy originated in Africa, extended to Asia and Europe, and arrived in the Americas during European colonization and the African slave trade. Due to colonization, the contemporary Colombian population is an admixture of Native-American, European and African ancestries. Because microorganisms are known to accompany humans during migrations, patterns of human migration can be traced by examining genomic changes in associated microbes. The current study analyzed 118 leprosy cases and 116 unrelated controls from two Colombian regions endemic for leprosy (Atlantic and Andean) in order to determine possible associations of leprosy with patient ancestral background (determined using 36 ancestry informative markers), *Mycobacterium leprae* genotype and/or patient geographical origin. We found significant differences between ancestral genetic composition. European components were predominant in Andean populations. In contrast, African components were higher in the Atlantic region. *M*. *leprae* genotypes were then analyzed for cluster associations and compared with the ancestral composition of leprosy patients. Two *M*. *leprae* principal clusters were found: haplotypes C54 and T45. Haplotype C54 associated with African origin and was more frequent in patients from the Atlantic region with a high African component. In contrast, haplotype T45 associated with European origin and was more frequent in Andean patients with a higher European component. These results suggest that the human and *M*. *leprae* genomes have co-existed since the African and European origins of the disease, with leprosy ultimately arriving in Colombia during colonization. Distinct *M*. *leprae* strains followed European and African settlement in the country and can be detected in contemporary Colombian populations.

## Author Summary

Contemporary Colombian population is an admixture of three ancestries: Native-American, European and African. Genetic studies of human ancestry have found associations with disease, likely due to the fact that microorganisms have accompanied humans during migrations. Taking these facts into account, we studied the effect of human ancestry, *Mycobacterium leprae* genotype and the geographical origin of our study population, on leprosy. We found correlations between ancestral composition and *M*. *leprae* genotype: an African component is higher in the Atlantic region and a European component is higher in Andean populations (p<0.05). An interesting connection was found between the ancestral composition and two principal types of *M*. *leprae* isolates: type C54 (of African origin) was more frequent in Atlantic region populations, and type T45 (of European origin) was more frequent in the Andean region, suggesting that human and bacterial genomes have co-existed since leprosy’s origins and that leprosy has circulated with human migration.

## Introduction

Leprosy is a chronic infectious disease caused by the bacterium *Mycobacterium leprae*, which affects skin, bones and peripheral nerves and results in irreversible deformities that stigmatize patients [1]. Leprosy is a millenary disease; it is possible that leprosy’s eradication has not been achieved due to important factors related to the non-controlled transmission of the disease [2–5]. In Colombia, leprosy is not considered a public health problem because its global prevalence is less than 1/10000 people [6, 7]. However, Colombia has several endemic regions where prevalence of the disease remains higher than 1/10000, such as in the Bolívar and Antioquia departments, regions included in the current study [8, 9].

The introduction of leprosy to the Americas occurred after the arrival of European conquerors and African slaves [10, 11]. Leprosy first appeared in Colombia along the Atlantic coast and subsequently spread to the hinterland along major rivers, such as the Magdalena. Historical reports indicate that the native population of Colombia did not suffer from leprosy [12–14], suggesting the introduction of the leprosy to Colombia occurred during European migration and the African slave trade. Because commensal and parasitic microorganisms are known to accompany human migration [15], the geographical history of migration can be traced by examining genomic changes in associated microbial agents. The genotyping of viral agents, such as hepatitis B, hepatitis G, polyomavirus JC, the fungus *Coccidioides immitis* and the Gram-negative bacterium *Helicobacter pylori* have provided compelling insights into patterns of human migration [15–18].

Leprosy is currently endemic to Africa and Asia, and was previously endemic to Europe until the Middle Age [19–21]. *M*. *leprae* has had considerable genomic conservation during the last 1000 years [20, 22, 23], making it is possible to compare extinct genotypes (e.g. strains obtained from skeletal remains) to those of existing strains. Such analyses allow for the determination of the predominant genotypes in areas where the disease has since disappeared [20, 21] and aid our understanding of the routes through which leprosy spread [10, 11, 20, 21]. The trade route between Europe and Asia known as the Silk Road may have contributed to the spread of leprosy in Europe, the Near East, the Far East and China [20, 21]. It was previously believed that leprosy was introduced to China from the Indian subcontinent. However, this hypothesis has not been confirmed by genotyping studies of *M*. *leprae* [10] nor has the idea that leprosy was brought to Europe from India by soldiers of Alexander the Great [10, 11, 24]. Therefore, it appears probable that colonialism, and not Asian migration through the Bering Strait, introduced leprosy to the New World [10].

Four *M*. *leprae* haplotypes have been identified to date [10], referred to as Single Nucleotide Polymorphism (SNP) types 1, 2, 3 and 4. SNP-type 1 is found in *M*. *leprae* strains from Asia, the Pacific region and eastern Africa, SNP-type 2 in isolates from Ethiopia, Malawi, Nepal, North India and New Caledonia, SNP-type 3 in strains from Europe, North Africa and the Americas and SNP-type 4 in strains from West Africa and the Caribbean [10]. Based on SNP [10, 11] and Variable Nucleotide Tandem Repeat (VNTR) typing [25], *M*. *leprae* strains from Colombia and Brazil are associated with SNP-types 1 to 4 [26, 27]. In Colombian SNP-type 3 strains, the allelic combination of the minisatellite loci 27–5 and 12–5 are 4–5 (in 4 and 5 copies, respectively), suggesting European origin. However, in other strains, the allelic combination of loci 27–5 and 12–5 are 5–4, frequently related to SNP-types 2 and 4 [26], suggesting African origin. The reversal in copy number of the 27–5 and 12–5 alleles suggest that SNP-type 4 South American haplotypes are not direct descendants of the local SNP-type 3, but instead diversified from global strains of types 1, 2 and 3 [26, 27].

Drawing from the reports mentioned above, VNTR markers can be used to identify local *M*. *leprae* subtypes. A second type of genomic marker, identified during drug resistance surveys, appears to be associated with SNP-type 3 strains [28–30], which agrees with the possible separation of the American SNP-type 3 strain indicated by alternative 27–5 and 12–5 alleles. This second marker is a C/T SNP in the gyrA gene (codon 99)—the ‘T’ allele is found only in American SNP-type 3 strains while the ‘C’ allele is found in all other strain types, including SNP-type 4. This gyrA SNP was originally classified as ‘non-informative’ for phylogenetic studies. However, recent reports found the same gyrA SNP in *M*. *leprae* strains from other regions (SNP7614 to indicate its genomic position), suggesting American SNP-type 3 strains are a branch of *M*. *leprae* that separated from other existent worldwide strains [30, 31].

The relationship between host-pathogen (e.g. *M*. *leprae* genotype with human genetic ancestry) can provide information about the origins of leprosy and its transmission in Colombia. The Colombian population is an admixture of three ancestral backgrounds: Native-American, African and European [32, 33], allowing the Colombian population to be used to identify risk genes for complex diseases that differ between parental groups [33]. Populations with a recent history of ancestral mixture [20 generations or less) are ideal for implementing genetic mapping with technology developed for massive typing and Ancestry Informative Markers (AIMs); new statistical methods allow us to analyze the mixed state dynamics in a population [34–36]. Currently, there are several AIM databases that distinguish the ancestral origin of continental populations and some regional subpopulations [32, 37].

Ancestral genetic composition influences susceptibility and/or resistance to numerous diseases such as hypertension, obesity and diabetes [34, 38–42]. Further, ancestral genetic composition is associated with the immune response to the infectious diseases dengue and leprosy [43–49]. However, data on how the mixture of ancestries influences the susceptibility to leprosy and its effect on the clinical severity of the disease is currently unavailable. In this study, we found significant differences between the ancestral genetic composition of Colombian populations in the Atlantic and Andean regions—differences related to the history of colonization in the country—and the dominant strains of leprosy that affect each population. Both European settlers and African slaves arrived in the Atlantic region. However, European settlers preferred residence in cooler areas and occupied the Andean region, while Africans populated the coast. Our findings show a correlation with ancestral genetic makeup of the people inhabiting these regions and the *M*. *leprae* haplotypes that infect patients.

## Materials and Methods

### Study population

This study was performed in two geographic regions: the Antioquia and Bolivar departments. The Antioquia department, located in the Andean region, has a leprosy prevalence of <1/10000. The Bolivar department, located in the Atlantic region, has a prevalence of >1/10000. We included 118 leprosy cases and 116 unrelated household contact controls as reported by national and local leprosy control programs in a period of 2007–2013. To describe geographical origin, we refer to these departments simply as the Andean region and the Atlantic region. Leprosy diagnosis was performed according to Colombian Ministry of Health guidelines, which were adapted from WHO leprosy guidelines: http://www.minsalud.gov.co/DocumentosyPublicaciones/GUIA DE ATENCIÓNDELEPRA.pdf.

The leprosy group was composed of 97 multibacillary (MB) and 21 paucibacillary (PB) leprosy patients, under treatment or post treatment. Bacillary index and clinical exams classified leprosy according to Ridley and Jopling criteria [50, 51]. Tuberculoid leprosy (TT) was defined as a PB patient with 1 to 5 skin lesions, hypo- or hyper-pigmented macula, clearly defined, with hypoesthesia, at least one compromised peripheral nerve increased in size and localized pain [52–54]. Lepromatous leprosy (LL) was defined as an MB patient with more than 5 skin lesions, nodules, plaques or papules, loss of sensitivity and several compromised peripheral nerves [52–54].

The control group was constituted by 116 household contacts (HHC) > 2 years of age who resided in the same house for ≥ 6 months with the index case. Blood relation with the index case, suspected skin lesions, loss of sensitivity, and pregnancy were considered exclusion criteria. The 116 controls were composed of spouses, friends, sons and daughters in law of the index case, all without exclusion criteria.

#### Ethical considerations

This study proposal was approved to the ethical committee of the Colombian Institute of Tropical Medicine—CES University, which classified this project as of minimal risk to volunteers, according to the resolution 8430 of 1993 of the Minister of Health of Colombia:

http://www.minsalud.gov.co/Normatividad/RESOLUCION%208430%20DE%201993.pdf. Volunteers were informed of the purpose of this study and signed a consent form.

### Clinical samples

DNA was extracted from whole blood of experimental and control groups using the published technique [55]. Purified DNA was quantified and the concentrations set between 0.02 and 1.5 μg/μl.

### Ancestry Informative Markers (AIMs)

We selected 36 AIM markers informative for Latin-American populations [56–59]. The 36 AIM markers are considered sufficiently informative variants, given that the difference in allele frequencies is higher than 0.30 between any of the parental groups [56–59]. The average delta for the selected panel was 0.435, 0.490 and 0.340 for African/European, African/Native-American and European/Native-American, respectively. Characterization of these markers was performed by PCR of the AIM containing region; primers sequences are shown in S1 Table. Genotypes were resolved by TAQMAN PCR-RFLP (in the case of SNPs) and by differences in the fragment size amplified (in cases of an IN/DEL) using an ABI Prism 310 Genetic Analyzer (Applied Biosystems). Determination of genotypes was performed using GeneMapper v4.0 (http://www.appliedbiosystems.com/absite/us/en/home/support/software/dna-sequencing/genemapper.html)

Allele and genotype frequencies for the AIMs were calculated with PLINK version 1.07 (http://pngu.mgh.harvard.edu/~purcell/plink/download.shtml). The Hardy-Weinberg equilibrium (HWE) was calculated using GENEPOP v4.0 (http://genepop.curtin.edu.au/). The significance of the deviations from HWE was assessed with Chi square tests of independence. Markers that had a p value of less than 0.05 in the control group were considered out of HWE and were not included in the calculation of indices for individual ancestry or association analyses. To determine the significance of the analysis controlled for the number of tests, a Bonferroni correction was performed. Four of 40 AIMs tested were not in HWE and were not included in our analyses. See S1 Table for the 36 AIMs used in the analysis, the allele frequencies in the ancestral populations and the discriminating power.

### Individual and population admixture proportions

We estimated admixture proportions for individuals (per volunteer), for the Andean and Atlantic population and for the total African/European/Native American population using 36 highly informative SNPs with the Admixmap V.3.8 program [36].

### Analysis of association

To evaluate the association of categorical variables with disease status, Chi square tests of independence were performed in SPSS (Statistical Package for the Social Sciences v21):

http://www-03.ibm.com/software/products/es/spss-stats-pro).

### *M*. *leprae* genotyping

The genotypes of 166 *M*. *leprae* strains reported in Cardona-Castro et al [28] were analyzed using the Network program (http://www.fluxus-engineering.com/sharenet.htm) and evaluated according to geographical origin. Briefly, *M*. *leprae* genotyping was based on the detection of the number of copies of two Variable Number Tandem Repeats (VNTRs) [25,26, 60], the minisatellite loci 27–5 and 12–5, SNP types 1–4 and the SNP (C/T) in gyrA (SNP7614) [28].

For analytical purposes, three markers formed a genotype—the first component of the letter C and T correspond to SNP7614, the second corresponds to the number of copies of the VNTR marker 27–5 and the third is the number of copies of 12–5. Interpreted as: gyrA / 27–5 / 12–5 [28].

### Geo-referencing methods

Residential location of the study population (latitude and longitude) was recorded by a Garmin eTrex Vista HCx instrument in the decimal degrees units system.

Location of genotypes: A vector map for viewing and grouping the geographical coordinates of each *M*. *leprae* genotype (according to different eco-geographical regions of Colombia) was generated using QGis version 2.8. The layers used (Colombia, eco-regions and sampling points) were created with the latitude and longitude in the WGS84 reference ellipsoid. The areas of greatest density points are highlighted in boxes.

Geospatial analysis of human ancestral composition. In order to assess the geographical context of the spatial variation of the ancestral composition, we mapped the origin of the volunteers that participated in this study. Based on the geographical coordinates of that origin, we carried out an interpolation of every racial mixture (African, European, Amerindian), taking the geographical region of these sources into account. The interpolation was carried out based on the geo-statistical method. The interpolator used was kriging ordinary, and its discretization was done on 2x2 blocks. The result of each ancestry kriging was validated with the cross validation method (cross-validate). For comparison purposes, each map includes experimental semivariogram values and their adjustment to the theoretical semivariogram.

The theoretical semivariogram is recognized by the following formula [61, 62]:

γ(h)=1/2nΣ[Z(xi)-Z(xi+h)]2

Where: h = distance between the pairs and n = number of pairs.

Z(xi) = the location and value of the sample.

**Fig 1:**
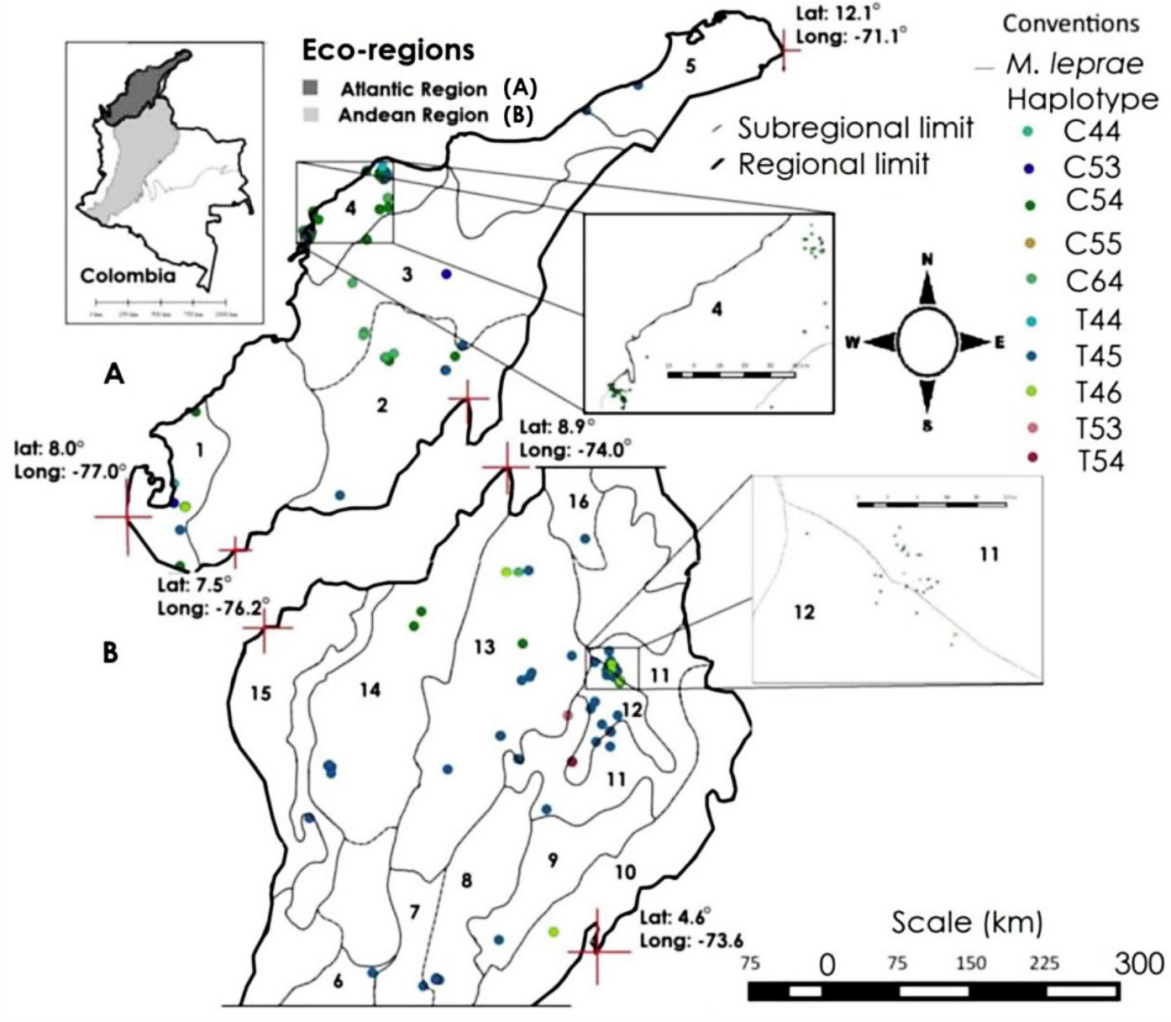
a. b. Geographical distribution of *M*. *leprae* genotypes in the Atlantic and Andean regions, respectively.

The application of the correlation function, subsequent kriging and mapping spatial variation was carried out with GS+ software, version 10.0. In advance, basic statistics were applied to verify normality of the data. The resulting maps were exported to the QGis GIS software, version 2.8, for vectorization and adjust to the used Datum (WGS 84). (Fig 1).

## Results

### Leprosy patients and controls

LL was the most frequent clinical diagnosis in our leprosy study group. The distribution of leprosy cases by region were as follows: 66% of patients (n = 78) were from the Atlantic region and 34% (n = 40) were from the Andean region. These percentages correspond to the frequency and geographical distribution of leprosy cases previously reported for Colombia [6]. The leprosy group consisted of 75.4% males and 24.6% females (a male/female ratio of 2.87/1). The control group consisted of 32.7% males and 67.3% females. The composition of the control group (a male/female ratio of 0.49/1) resulted from sampling—females were more available than males at the time of sample collection.

### Geographical context of genotypes

Ten different genotypes were distributed in at least 16 ecological subregions and two ecoregions of Colombia: Five subregions of the Atlantic region (the Colombian Caribbean region), and 11 sub-regions of the central and northern Colombian Andean region (Fig 1A and 1B). We detected at least three unique genotypes from the Andean region: T54, located only in the Chicamocha and Suarez river canyon; T53, located on the border between the Magdalena Medio and Santandereana Mountain subregions; C44, located in the boundary between the Canyon of Chicamocha and Suarez rivers in Santander Mountain (Fig 1A and 1B). In the Atlantic region only one unique genotype was observed: C53, located in the Gulf of Urabá and Mompox Depression. The most common genotypes at two eco-regions were: T44, T45 (perhaps the most dispersed genotypes), T46, C54, C55, and C64.

### Ancestral genetic composition

The individual ancestral composition of leprosy cases and controls are shown in S2 Table.

**Fig 2:**
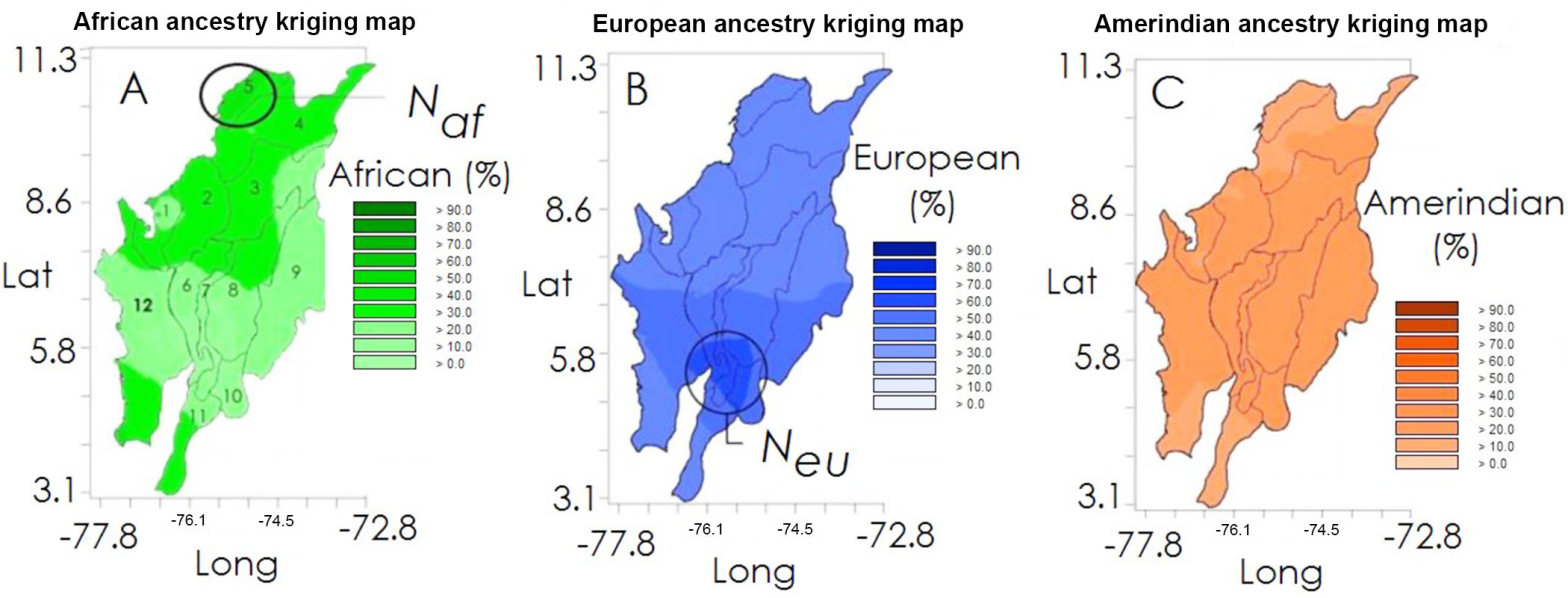
(A) Geographic distribution of African (green), (B) European (blue), (C) Amerindian (orange) ancestry based on individual estimates. To facilitate comparison, color intensity transitions occur at 10% ancestry intervals for all maps. Maps were obtained using Kriging interpolation (see Materials and Methods).

Significant differences were observed between the ancestral genetic composition among the study population from the Atlantic and Andean regions, Fig 2A–2C. In the Andean region, the European component of leprosy patients and controls was found to be higher than the African and Native American components (p<0.05). In contrast, the African component of leprosy patients and controls was higher in the Atlantic region (p<0.05).

**Fig 3:**
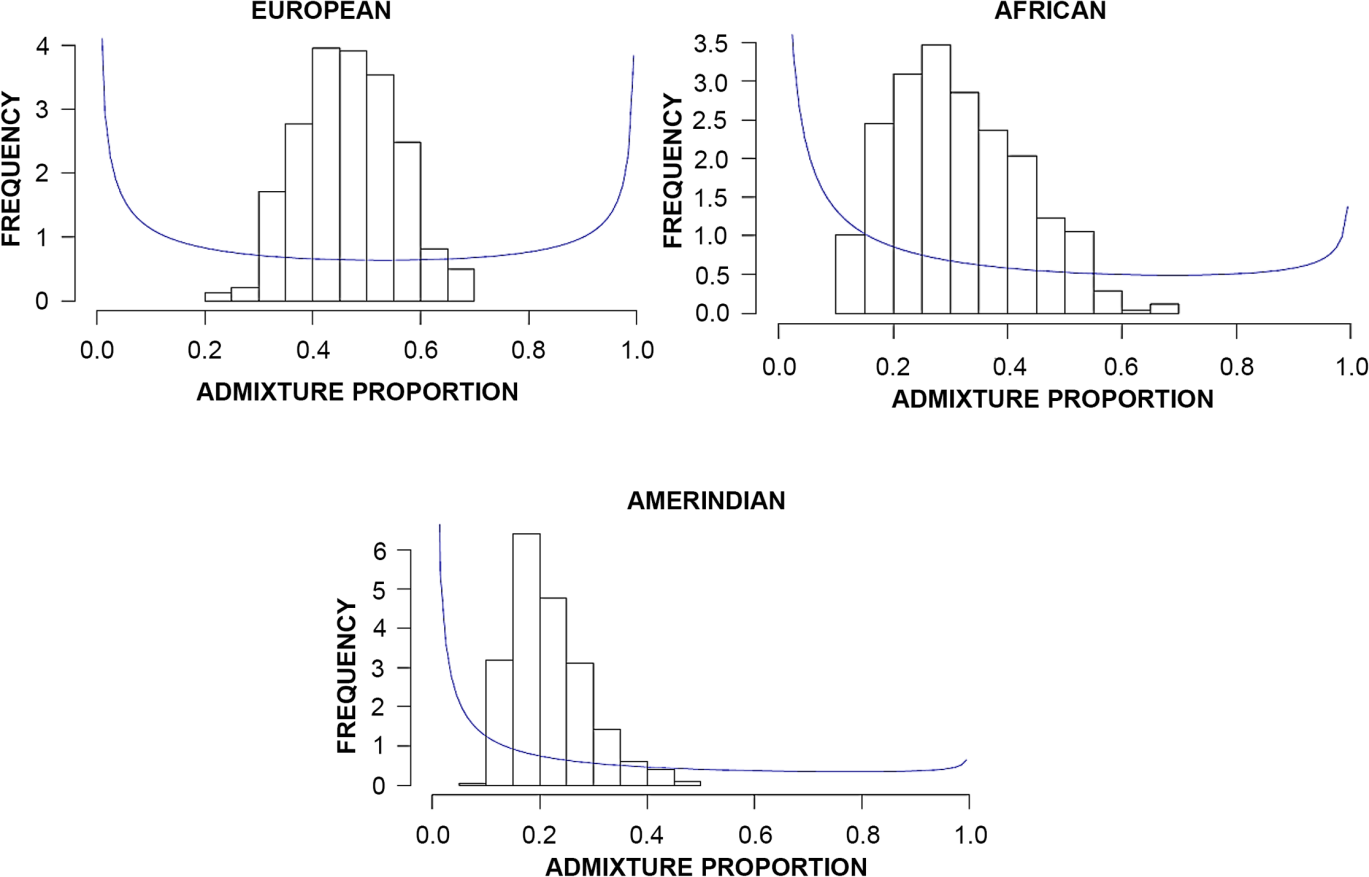
Dirichlet distributions of the ancestral components of the study population. The European component had a normal distribution and consisted of 40–60% of the study population. The African component had a non-normal distribution and consisted of less than 30% of the study population. The Native American (Amerindian) component had a non-normal distribution and consisted of 20% of the study population.

Dirichlet distributions of the proportion of European, African and Native American ancestral components in the study population are shown in Fig 3. Comparisons of medians of the ancestral genetic component by geographical region of origin (Mann-Whitney U-tests) are shown in Table 1.

**Table 1:** Comparison of medians of ancestral components according to the geographical origin of the population, Andean vs. Atlantic regions (Mann-Whitney U-test).

Region/ Ancestral Component	Andean region	Atlantic region	P value
**European**	0.523	0.466	0.0000001
**African**	0.245	0.338	0.0000001
**Native-American**	0.226	0.195	0.00002

### Geospatial analysis of human ancestral composition

#### African ancestry

Interpolation results show that there is spatial dependence (i.e. the distance from which two observations are independent; represented by "Ao" in the Fig 2A–2C) between values in a distance of approximately 366 km (Ao = 3.3°). This suggests that African ancestry is best represented in the Atlantic region, with ancestry percentage values ranging between 30 and 60% in most of its sub-regions, and a concentration of the population with 60–70% African ancestry in the Magdalena River delta. It should be noted that at the time of the Spanish conquest, the Magdalena River was the gateway of African slave labor (Fig 2A).

#### European ancestry

The spatial dependence observed for this ancestry is more than twice that found in African ancestry. Further, it has a range of ~770 km; it practically covers the entire study area (Fig 2B). However, the highest percentages of European ancestry (> 70%) in the study population was located in the central Andean region (especially south of the Middle Magdalena River subregion), the Antioquia Mountain and northeast Andean Cordillera Occidental sector.

#### Amerindian ancestry

The distribution of this ancestry beyond that distance can be considered random (it presents a more or less uniform spatial distribution) (Fig 2C). In terms of area, we observed that most of the villagers of the Andean and Atlantic regions had between 30 and 40% of this ancestry, except in the previously mentioned areas where the largest ancestries were African and European.

### Leprosy and the genetic ancestral component

Mann-Whitney U tests were performed to determine possible associations of the ancestral composition between the leprosy and control groups. The average ancestral components for each group showed no association with leprosy (p>0.05).

We also compared the ancestral genetic composition between leprosy and control groups according to their region of origin. We found no significant differences in ancestral genetic composition between the leprosy and control groups (p>0.05; Table 2).

**Table 2:** Comparison of means for each ancestral component by geographical region for the leprosy and control groups.

Ancestral component/ region	Leprosy Cases	Controls	P value
	average / variance	average/variance	
Atlantic region			
European	0.456 / 0.007	0.451 / 0.008	0.72
African	0.355 / 0.011	0.348 / 0.013	0.74
Amerindian	0.188 / 0.004	0.199 / 0.005	0.34
Andean region			
European	0.508 / 0.011	0.541 / 0.009	0.15
African	0.253 / 0.013	0.216 / 0.001	0.14
Native-American	0.239 / 0.007	0.243 / 0.009	0.85

### Ancestral genetic composition of MB vs PB leprosy patients

Averages of ancestral components of MB and PB leprosy patients were compared. We found no significant differences between the ancestral component and leprosy type in this population (Table 3).

**Table 3:** Comparison of average ancestral component in patients diagnosed with leprosy type MB vs. PB (Mann-Whitney U-tests).

Ancestry/type of leprosy	PB*	MB**	P value
African	0.303	0.325	0.695
European	0.481	0.467	0.611
Native-American	0.214	0.204	0.540

*PB: paucibacillary patients included tuberculoide leprosy (TT), borderline tuberculoide leprosy (BT), and indeterminate leprosy (IL).

**MB: multibacillary patients, included lepromatous leprosy (LL), borderline lepromatous (BL).

### Network haplotypes of *M*. *leprae*

**Fig 4:**
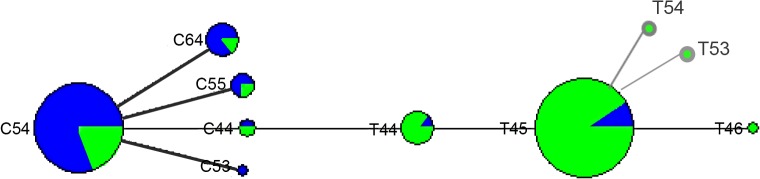
Haplotype network of the genotyped strains SNP7614, VNTR 27–5 and VNTR 12–5.

For each genetic marker, alleles were analyzed for network configuration. Two main haplotypes, C54 (African origin) and T45 (European origin) were observed (Fig 4). The C54 haplotype was primarily distributed in the Atlantic region (80.4%) and the T45 haplotype was principally distributed in the Andean region (90.9%) (p = 1x10^–8^). The T44 haplotype appears to connect the two main strains. Migration of patients between different Colombian regions may have led some strains from one region to another. This is confirmed in patients who originated from a site that differed from their involvement in the study (S1 Fig). It is possible that the two principal genotypic strains found in Colombia C54 (type1) and T45 (type 2) were transmitted and segregated from the Atlantic and the Andean region, respectively (Fig 4). We hypothesize that the most common haplotypes, C54 and T45, are distributed in Colombian regions where the ancestral origin of the patients are related with the strains origin (strain origin inferred by the SNP type 2, 4 and 3). Further, we suggest that the C54 and T45 haplotypes are the origin of the other haplotypes we observed.

Colors represent the frequency of the strains in regions: Blue = Atlantic region, Green = Andean region.

As a complementary illustration, the geographic distribution of every haplotype detected by the geographical origin of the strain is shown in Table 4.

**Table 4:** *M*. *leprae* haplotypes SNP7614, VNTR 27–5 and 12–5 by geographical origin.

Haplotype/Region	Andean N (%)	Atlantic N (%)	P value/total
C44	1 (50%)	1 (50%)	—
C53	2 (67%)	1 (33%)	—
C54	8 (15,4%)	44 (84,6%)	1X10^–8^
C55	1 (25%)	3 (75%)	—
C64	1 (7,7%)	12 (92,3%)	<0,05
T44	6 (85,7%)	1 (14,3%)	<0,05
T45	69 (90,8%)	7 (9,2%)	1X10^–8^
T46	6 (85,7%)	1 (14,3%)	<0,05
T53	1 (100%)	0	0.317
T54	1(100%)	0	0.317
Total	96	70	166

## Discussion

In this study, we attempted to correlate previously reported *M*. *leprae* genotyping data [28] with the ancestral genetic composition of two Colombian populations. The geographical spread of two major *M*. *leprae* strains identified as having European and African origins are distributed according to the ancestral genetic composition of the population. In the Andean region, the *M*. *leprae* strain of European origin circulates in the population where the European ancestral component is higher than the African and Native American components (p<0.05). In contrast, *M*. *leprae* of African origin circulates in the Atlantic region where the African component is higher (p<0.05). Our findings are supported by two previous studies of leprosy in Colombia [26, 28] and suggest that the African and European origins of *M*. *leprae* strains are related to the ancestral genetic composition of the Colombian population; strains that originated in Africa and Europe were brought to Colombia during colonization and are now circulating in populations where African and European colonizers originally settled (reflected in the genetic composition of contemporary Colombians),

It has previously been suggested that leprosy originated in Central Asia/East Africa and subsequently migrated with humans [10]. This hypothesis is based on the analysis of *M*. *leprae* SNP types 1 to 4. Analysis of Colombian and Brazil *M*. *leprae* strains suggested that the South American SNP type 4 did not descend from local SNP type 3, but instead diversified from global strains of SNP types 1, 2 and 3 [26; 27]. Additionally, a second genomic marker—a C/T SNP in the *gyrA* gene, codon 99—was identified during drug resistance surveillance [29, 30]. This SNP (denoted as SNP7614 to indicate position in *M*. *leprae* genome) appears to associate with type 3 strains; the ‘T’ allele is found only in American SNP type 3 strains while the ‘C’ allele is found in all other regions and types. Thus, the *gyrA* SNP demonstrates that American SNP type 3s are a branch of *M*. *leprae* that separated from other extant global strains [30]. We currently know of at least 40 VNTR loci and several hundred SNPs that aide in *M*. *leprae* genetic mapping. Because the rates of incidence, the origin of populations and the history of leprosy differ by region, VNTR and SNP allelic diversities have been shown to be informative of local *M*. *leprae* subtypes [60, 63], we used these facts to examine *M*. *leprae* strains in two Colombian regions endemic for leprosy.

While our results suggest that leprosy was introduced to Colombia and subsequently spread by European colonization and the introduction of African slaves, we cannot rule out additional factors that may have played a role in the distribution of *M*. *leprae* genotypes we observed. It is unclear if any one *M*. *leprae* strain is more virulent than another. Public health conditions are poor in both geographical areas surveyed, which may have contributed to the populations’ susceptibility to the disease. The clinical outcome of leprosy and the spectrum of the disease has been shown to be directly associated with the immune response of the patient [47, 48, 51]. Lastly, migration is common in Colombia due to several social facts (e.g. the guerrilla war). Some of the *M*. *leprae* genotypes we found more frequent in the Andean region were also found to infect people of the Atlantic region. To address this and to explain the distribution of the *M*. *leprae* genotypes we observed, we collected data from every patient regarding their personal geographical movements—S1 Fig shows the place of diagnosis of each patient and their place of the origin.

The settlement of Colombia by European and African peoples led to a population that is today the product of three ancestries: Native-American, European, and African [33]. Our results suggest that European and African ancestors brought leprosy to Colombia, highlighting the relationship between the origins of the disease in Colombia, the ancestral genetic composition of the population and the origin of *M*. *leprae* strains [10, 28]. It can be inferred that these *M*. *leprae* strains genetically differ according to geographical origin, a fact that may be associated with the ancestral origin of the Colombian population.

There is evidence that leprosy was not present in native Colombian populations [12, 64, 67]. Historical documents suggest that leprosy arrived in Colombia through the city of Cartagena (Bolívar department) with reports of the disease in Cartagena from 1598 to 1608 [64]. One of the first leprosy patients on record is the founder of Santa Fe de Bogota, Gonzalo Jimenez de Quesada [12, 14, 64, 65]. Leprosy then spread into Colombia by the Magdalena River—the disease was first reported inland in Mompox City and Socorro in 1745, coinciding with the establishment of Spanish and African settlements there [64–66]. Around 9.2 million African slaves were brought to the Americas [65–66], thousands of which were suffering from leprosy, resulting in the founding of the first Colombian leprosarium [65,69]. However, settlements on the Atlantic and Pacific Regions were the most common [66], likely influencing the ancestral genetic composition of these regions.

### Gender ratio of leprosy

The frequency of leprosy by gender corresponds to a ratio of 2.87 (2.87 male /1 female). This male/female ratio (3/1), has been widely reported in the literature [1]. The cause of this frequency is unclear. The composition of the control group in the current study, a male/female ratio 0.55/1, was due to the sampling. However the gender composition of the control group does not affect the ancestral composition, since we did not test chromosome X and Y markers.

### Ancestral genetic composition and leprosy

The results reported here highlight the ancestral genetic similitude of MB and PB leprosy patients, which show no significant difference in leprosy types and ancestral background. The non-association between ancestral component and leprosy suggests that all clinical outcomes of leprosy are possible, regardless of racial ancestry. This finding may be related to the presence of LL, BL (MB) and TT, IL, BT (PB) leprosy in both European and African ancestral populations [3,4,70].

### Conclusions

Our results show that the ancestral genetic composition in populations of the Atlantic region differ with respect to populations of the Andean region—we found a higher proportion of an African component in the Atlantic region and a higher proportion of a European component in the Andean region, findings consistent with the history of settlement in Colombia [65, 66]. European settlers were located mainly in mountainous areas, now referred to as the Andean region, while Africans arrived in greater numbers in the Atlantic region. *M*. *leprae* strain origin correlates with the distribution of genotypes found in the Atlantic and Andean regions—*M*. *leprae* strains of African origin are transmitted more frequently in the Atlantic region while *M*. *leprae* strain of European origin are distributed more frequently in the Andean region, suggesting that human ancestral genetic composition and *M*. *leprae* origin are related. These results support the findings of Monot *et al* [10, 11] regarding the origin of leprosy in the Americas and are applicable to Colombian populations in leprosy endemic regions. Both genomes, from the affected population and the causative agent, are genetically correlated—they have been maintained together since their African and European origins and have accompanied each other during times of human migration. When colonization brought Europeans and slaves to Colombia, leprosy appears to have arrived with them, as there are no accounts of the disease in the native population prior to their arrival [12, 13, 68, 69]. It is intriguing to suggest that the absence of leprosy in Colombia may be due to the ancestral Native-American component acting as protection against leprosy (OR = 0.5, CI 95% = 0.330–0.982, p = 0.043). However, this result should be examined in a future study of larger sample size.

## Supporting Information

S1 Table Ancestral Informative Markers (AIMs), frequency and delta of frequencies in the three ancestral populations, and primers used to determine the ancestral composition of the population. (DOCX)

S2 Table Individual ancestral composition of leprosy cases and controls according to leprosy diagnosis, age, sex, and region of origin. (DOCX)

S1 Fig Geographic origin of patients and place where the leprosy diagnosis was performed. (TIF)
