# Supplementary material for: Human Genetic Ancestral Composition Correlates with the Origin of Mycobacterium leprae Strains in a Leprosy Endemic Population
Source: PLoS Negl Trop Dis. 2015 Sep 11;9(9):e0004045. doi: 10.1371/journal.pntd.0004045 (PMC4567314; doi:10.1371/journal.pntd.0004045)
Supplement: S1 Table — (DOCX) [file pntd.0004045.s001.docx]

**Supplementary Table 1.** Ancestral Informative Markers (AIMs), frequency and delta of frequencies in the three ancestral populations, and primers used to determine the ancestral composition of the population.

| AIM | Chrom* | African (AF)+ | European (EU)+ | Amerindian (AM)+ | d l AF-EU l & | d l AF-AM l& | d l EU-AM l& | Primer F (5’-3’) | Primer R (5’-3’) |
| --- | --- | --- | --- | --- | --- | --- | --- | --- | --- |
| MID1752 | 1 | 0.560 | 0.290 | 0.920 | 0.270 | 0.360 | 0.630 | TGTTTGTACCTTCCAAGTCTCT | AGATTGACATTCCTTCCACA |
| FY-NULL | 1 | 0.001 | 0.998 | 1.000 | 0.997 | 0.999 | 0.002 | GTAAAATCTCTACTTGCTGGAAG | CCATGGCACCGTTTGGTTCAGG |
| AT3 | 1 | 0.858 | 0.282 | 0.061 | 0.576 | 0.797 | 0.221 | CCACAGGTGTAACATTGTGT | GAGATAGTGTGATCTGAGGC |
| GC-1F | 4 | 0.853 | 0.156 | 0.339 | 0.697 | 0.514 | 0.183 | AGATCTGAAATGGCTATTATTTTGC | GGAGGTGAGTTTATGGAACAGC |
| GC-1S | 4 | 0.931 | 0.393 | 0.458 | 0.538 | 0.473 | 0.065 |  |  |
| MID52 | 4 | 0.740 | 0.840 | 0.140 | 0.100 | 0.600 | 0.700 | GCACAGGTGTTTTAGAGGC | GTTCCAGTTGTTGGTGTGAC |
| MID817 | 5 | 0.960 | 0.650 | 0.130 | 0.310 | 0.830 | 0.520 | ATTACCGGAACACATTCTGA | CCTACATCCAACAGAAGGTG |
| MID1039 | 5 | 0.980 | 0.270 | 0.830 | 0.710 | 0.150 | 0.560 | CGTTTCATCTCTTTGGGTTA | GCTTTCTTCATTCCTTCACA |
| MID1358 | 5 | 0.800 | 0.060 | 0.040 | 0.740 | 0.760 | 0.020 | GTTTTGGGAATTTAGGTTTTG | AGACGCCAGGAATTTTCTAT |
| MID856 | 5 | 0.660 | 0.150 | 0.690 | 0.510 | 0.030 | 0.540 | AACATGGGAACTGCTCATTA | TATTGTGCTCATTTTCTGGG |
| MID944 | 5 | 0.890 | 0.390 | 0.950 | 0.500 | 0.060 | 0.560 | TCAGTAAAAGGGTTTCCTTGT | GTAAGCAGCCTGGATTACAA |
| MID108 | 6 | 0.580 | 0.320 | 0.040 | 0.260 | 0.540 | 0.280 | CTCCTCCTCATCCAAAAATT | GCATCTGTTGCCATTGTT |
| MID154 | 20 | 0.820 | 0.250 | 0.140 | 0.570 | 0.680 | 0.110 | GGCTCTGACTGAGAAACTGA | AACAGGCAATCCTCCTAAGT |
| MID2062 | 6 | 0.410 | 0.290 | 0.930 | 0.120 | 0.520 | 0.640 | GGCCTGCATGATAAATAGAA | GAAGCCAGAACAATGAAAGA |
| MID1066 | 7 | 0.840 | 0.280 | 0.260 | 0.560 | 0.580 | 0.020 | TCTTGGGACTCAGAGTTCAG | CAGAAACCAAGGTGAAAGTG |
| LPL | 8 | 0.971 | 0.492 | 0.442 | 0.479 | 0.529 | 0.050 | AGGCTTCACTCATCCGTGCCTCC | TTATGCTGCTTTAGACTCTTGTC |
| MID1780 | 11 | 0.740 | 0.230 | 0.690 | 0.510 | 0.050 | 0.460 | TGACTTCAGTGTCTGCTGAA | ACACTTGCAGAGAGCTTTGT |
| DRD2 | 11 | 0.135 | 0.670 | 0.045 | 0.535 | 0.090 | 0.625 | CCTCTGAGGCTTACTGTCTG | AAAACTAGGGAGGGTCAGAG |
| APOA | 11 | 0.420 | 0.925 | 0.977 | 0.505 | 0.557 | 0.052 | AAGTGCTGTAGGCCATTTAGATTAG | AGTCTTCGATGACAGCGTATACAGA |
| MID1723 | 12 | 0.900 | 0.180 | 0.150 | 0.720 | 0.750 | 0.030 | CTTCAAACTATGGTCTTCAAAAA | GAGCAAAAGTGTAATTTCCCT |
| RB2300 | 13 | 0.926 | 0.315 | 0.175 | 0.611 | 0.751 | 0.140 | CAGGACAGCGGCCCGGAG | CTGCAGACGCTCCGCCGT |
| MID2269 | 13 | 0.900 | 0.400 | 0.900 | 0.500 | 0.000 | 0.500 | TTTCTCCACTGCGTTCAGTA | ACCAGAGTGGCTACTTTTGG |
| OCA2 | 15 | 0.115 | 0.746 | 0.488 | 0.631 | 0.373 | 0.258 | CTTTCGTGTGTGCTAACTCC | ACCTCTAGCATGGTTCTTGGGC |
| MID818 | 16 | 0.090 | 0.780 | 0.980 | 0.690 | 0.890 | 0.200 | TAGAGCCAGTTAGAGGGAGG | ACTTCAGTCGTCACTCCATC |
| SB19.3 | 19 | 0.415 | 0.903 | 0.645 | 0.488 | 0.230 | 0.258 | TCTAGCCCCAGATTTATGGTAACTG | AAGCACAATTGGTTATTTTCTGAC |
| PV92 | 16 | 0.225 | 0.152 | 0.792 | 0.073 | 0.567 | 0.640 | GGATCTCAGGGTGGGTGGCAATGCT | GAAAGGCAAGCTACCAGAAGCCCCAA |
| NBC4 | 2 | 0.513 | 0.683 | 0.947 | 0.170 | 0.434 | 0.264 | CATTCCACCCTGTCAGCATT | GCTTTGGAAGTAGGCAGGTTAC |
| Ya5ACA1100 | 3 | 0.450 | 1.000 | 1.000 | 0.550 | 0.550 | 0.000 | GCATCCTACAAAGCCATT | GCCTGGGCAATAATTTTCAA |
| DCP1-ALU/ACE Alu Indel | 17 | 0.349 | 0.346 | 0.730 | 0.003 | 0.381 | 0.384 | CTGGAGACCACTCCCATCCTTTCT | GATGTGGCCATCACATTCGTCAGAT |
| Ya5NBC150 | 19 | 0.300 | 0.924 | 0.944 | 0.624 | 0.644 | 0.020 | AAATGGAGACACAGAGGTGTAAAGA | CCCAAACTGCATATTTAAAGGGTAG |
| Ya5ACA636 | 1 | 0.975 | 0.579 | 1.000 | 0.396 | 0.025 | 0.421 | ACTTAAAGTGTTCAGCGGGG | ATTTTCCACCAACCAGGACA |
| Ya5_435 | 17 | 0.200 | 0.400 | 1.000 | 0.200 | 0.800 | 0.600 | CTGGCGACTAAGGTGAAAGC | AAAAGGTAATCCCTCTATCCTCTTG |
| D1 Alu insertion | 3 | 0.256 | 0.011 | 0.500 | 0.245 | 0.244 | 0.489 | TGCTGATGCCCAGGGTTAGTAAA | TTTCTGCTATGCTCTTCCCTCTC |
| pAlu6-17534722 | 6 | 0.125 | 0.750 | 0.375 | 0.625 | 0.250 | 0.375 | TACCTCGATAGTCTCACTTC | GTGTTGGTAGTGAAGAGAGCCAAC |
| Ya5ACA1184 | 4 | 0.158 | 0.688 | 0.947 | 0.530 | 0.789 | 0.259 | TGGCTCTAATGACCAAAAGGA | CCCAGGTGATTCATTCCATC |
| Ya5ACA1611 | 7 | 0.211 | 0.611 | 1.000 | 0.400 | 0.789 | 0.389 | TTTTGGTAAAGATGCCACAGAA | TCCTAAACATAATACGTACAGGTGA |

***Chromosome where the AIM is located. +Frequency in Africans, in Europeans, and in Native-Americans. &Delta of frequencies**
