# Supplementary material for: Human Genetic Ancestral Composition Correlates with the Origin of Mycobacterium leprae Strains in a Leprosy Endemic Population
Source: PLoS Negl Trop Dis. 2015 Sep 11;9(9):e0004045. doi: 10.1371/journal.pntd.0004045 (PMC4567314; doi:10.1371/journal.pntd.0004045)
Supplement: S2 Table — (DOCX) [file pntd.0004045.s002.docx]

**Supplementary Table 2.** Individual ancestral composition of leprosy cases and controls according to leprosy diagnosis, age, sex, and region of origin.

| ID | REGION | CASE DIAGNOSIS/  CONTROLS* | SEX | AGE | INDIVIDUAL ANCESTRAL COMPOSITION** | | |
| --- | --- | --- | --- | --- | --- | --- | --- |
|  |  |  |  |  | AFRICAN | EUROPEAN | NATIVE-AMERICAN |
| COL8 | ATLANTIC | LL-MB | MALE | 71 | 0.672 | 0.238 | 0.09 |
| COL1 | ATLANTIC | TT-PB | FEMALE | 36 | 0.447 | 0.428 | 0.125 |
| COL10 | ATLANTIC | TT-PB | MALE | 57 | 0.536 | 0.31 | 0.153 |
| COL15 | ATLANTIC | LL-MB | MALE | 87 | 0.304 | 0.527 | 0.169 |
| COL22 | ATLANTIC | TT-PB | MALE | 50 | 0.345 | 0.498 | 0.157 |
| COL24 | ATLANTIC | CONTROL | FEMALE | 45 | 0.524 | 0.258 | 0.218 |
| COL26 | ATLANTIC | LL-MB | FEMALE | 31 | 0.381 | 0.348 | 0.271 |
| COL27 | ATLANTIC | CONTROL | FEMALE | 20 | 0.381 | 0.428 | 0.191 |
| COL28 | ATLANTIC | LL-MB | MALE | 67 | 0.394 | 0.334 | 0.272 |
| COL29 | ATLANTIC | CONTROL | FEMALE | 45 | 0.303 | 0.502 | 0.195 |
| COL30 | ATLANTIC | CONTROL | MALE | 66 | 0.57 | 0.307 | 0.122 |
| COL31 | ATLANTIC | CONTROL | FEMALE | 67 | 0.559 | 0.322 | 0.119 |
| COL35 | ATLANTIC | LL-MB | FEMALE | 36 | 0.277 | 0.614 | 0.108 |
| COL36 | ATLANTIC | TT-PB | MALE | 40 | 0.519 | 0.358 | 0.123 |
| COL39 | ATLANTIC | CONTROL | FEMALE | 23 | 0.442 | 0.356 | 0.202 |
| COL45 | ATLANTIC | TT-PB | MALE | 35 | 0.309 | 0.48 | 0.211 |
| COL46 | ATLANTIC | CONTROL | MALE | 72 | 0.488 | 0.403 | 0.109 |
| COL51 | ATLANTIC | TT-PB | MALE | 70 | 0.354 | 0.454 | 0.192 |
| COL52 | ATLANTIC | CONTROL | FEMALE | 69 | 0.525 | 0.327 | 0.148 |
| COL57 | ATLANTIC | CONTROL | MALE | 23 | 0.493 | 0.338 | 0.168 |
| COL118 | ATLANTIC | LL-MB | MALE | 37 | 0.314 | 0.498 | 0.187 |
| COL120 | ATLANTIC | LL-MB | FEMALE | 25 | 0.243 | 0.515 | 0.242 |
| COL122 | ATLANTIC | TT-PB | FEMALE | 60 | 0.395 | 0.492 | 0.113 |
| COL134 | ATLANTIC | LL-MB | FEMALE | 71 | 0.106 | 0.709 | 0.185 |
| COL137 | ATLANTIC | LL-MB | MALE | 14 | 0.533 | 0.356 | 0.111 |
| COL140 | ATLANTIC | TT-PB | MALE | 32 | 0.439 | 0.417 | 0.145 |
| COL148 | ATLANTIC | LL-MB | MALE | 39 | 0.293 | 0.487 | 0.22 |
| COL153 | ATLANTIC | LL-MB | MALE | 50 | 0.199 | 0.423 | 0.378 |
| COL157 | ATLANTIC | BL-MB | FEMALE | 75 | 0.207 | 0.581 | 0.212 |
| COL159 | ATLANTIC | LL-MB | FEMALE | 39 | 0.696 | 0.214 | 0.09 |
| COL161 | ATLANTIC | TT-PB | MALE | 43 | 0.406 | 0.462 | 0.132 |
| COL162 | ATLANTIC | CONTROL | FEMALE | 40 | 0.253 | 0.547 | 0.2 |
| COL163 | ATLANTIC | TT-PB | FEMALE | 45 | 0.361 | 0.507 | 0.133 |
| COL165 | ATLANTIC | CONTROL | MALE | 13 | 0.518 | 0.352 | 0.13 |
| COL166 | ATLANTIC | CONTROL | MALE | 7 | 0.443 | 0.401 | 0.157 |
| COL167 | ATLANTIC | CONTROL | FEMALE | 30 | 0.411 | 0.446 | 0.143 |
| COL168 | ATLANTIC | CONTROL | FEMALE | 11 | 0.499 | 0.343 | 0.157 |
| COL171 | ATLANTIC | CONTROL | FEMALE | 58 | 0.413 | 0.404 | 0.184 |
| COL172 | ATLANTIC | TT-PB | MALE | 66 | 0.239 | 0.559 | 0.202 |
| COL173 | ATLANTIC | BB-PB | MALE | 48 | 0.438 | 0.406 | 0.156 |
| COL174 | ATLANTIC | CONTROL | FEMALE | 64 | 0.533 | 0.341 | 0.126 |
| COL175 | ATLANTIC | LL-MB | FEMALE | 64 | 0.348 | 0.482 | 0.17 |
| COL179 | ATLANTIC | CONTROL | FEMALE | 26 | 0.467 | 0.403 | 0.13 |
| COL180 | ATLANTIC | CONTROL | FEMALE | 22 | 0.58 | 0.304 | 0.115 |
| CON1 | ATLANTIC | LL-MB | MALE | 48 | 0.291 | 0.398 | 0.311 |
| CON2 | ATLANTIC | LL-MB | MALE | 78 | 0.377 | 0.485 | 0.138 |
| CON4 | ATLANTIC | LL-MB | MALE | 35 | 0.389 | 0.4 | 0.211 |
| CON7 | ATLANTIC | LL-MB | MALE | 38 | 0.403 | 0.402 | 0.194 |
| CON8 | ATLANTIC | LL-MB | MALE | 66 | 0.426 | 0.429 | 0.144 |
| CON9 | ATLANTIC | LL-MB | MALE | 48 | 0.376 | 0.429 | 0.195 |
| CON10 | ATLANTIC | CONTROL | FEMALE | 50 | 0.346 | 0.469 | 0.185 |
| CON11 | ATLANTIC | LL-MB | MALE | 66 | 0.239 | 0.47 | 0.291 |
| CON12 | ATLANTIC | CONTROL | FEMALE | 57 | 0.407 | 0.444 | 0.149 |
| CON13 | ATLANTIC | CONTROL | FEMALE | 41 | 0.217 | 0.52 | 0.264 |
| CON14 | ATLANTIC | LL-MB | MALE | 57 | 0.292 | 0.526 | 0.182 |
| CON15 | ATLANTIC | LL-MB | MALE | 88 | 0.526 | 0.368 | 0.106 |
| CON16 | ATLANTIC | LL-MB | MALE | 48 | 0.423 | 0.399 | 0.178 |
| CON17 | ATLANTIC | CONTROL | FEMALE | 38 | 0.486 | 0.353 | 0.161 |
| CON18 | ATLANTIC | CONTROL | FEMALE | 18 | 0.281 | 0.532 | 0.187 |
| CON19 | ATLANTIC | CONTROL | MALE | 11 | 0.393 | 0.468 | 0.139 |
| CON20 | ATLANTIC | CONTROL | MALE | 7 | 0.389 | 0.397 | 0.214 |
| CON21 | ATLANTIC | LL-MB | MALE | 58 | 0.563 | 0.313 | 0.124 |
| CON22 | ATLANTIC | LL-MB | MALE | 37 | 0.311 | 0.54 | 0.15 |
| CON23 | ATLANTIC | CONTROL | FEMALE | 35 | 0.531 | 0.329 | 0.141 |
| CON24 | ATLANTIC | LL-MB | FEMALE | 86 | 0.184 | 0.684 | 0.132 |
| CON25 | ATLANTIC | CONTROL | MALE | 38 | 0.23 | 0.592 | 0.178 |
| CON26 | ATLANTIC | LL-MB | MALE | 66 | 0.284 | 0.55 | 0.166 |
| CON27 | ATLANTIC | LL-MB | MALE | 54 | 0.32 | 0.47 | 0.21 |
| CON28 | ATLANTIC | CONTROL | FEMALE | 68 | 0.391 | 0.449 | 0.16 |
| CON29 | ATLANTIC | LL-MB | MALE | 53 | 0.219 | 0.495 | 0.286 |
| CON30 | ATLANTIC | LL-MB | FEMALE | 39 | 0.266 | 0.581 | 0.153 |
| CON31 | ATLANTIC | CONTROL | FEMALE | 24 | 0.208 | 0.578 | 0.214 |
| CON32 | ATLANTIC | LL-MB | FEMALE | 76 | 0.376 | 0.468 | 0.156 |
| CON33 | ATLANTIC | CONTROL | FEMALE | 48 | 0.493 | 0.374 | 0.133 |
| CON34 | ATLANTIC | LL-MB | MALE | 49 | 0.419 | 0.323 | 0.259 |
| CON35 | ATLANTIC | CONTROL | FEMALE | 16 | 0.535 | 0.337 | 0.128 |
| DSSA22 | ANDEAN | LL-MB | FEMALE | 64 | 0.519 | 0.346 | 0.135 |
| DSSA23 | ANDEAN | CONTROL | MALE | 55 | 0.122 | 0.689 | 0.188 |
| DSSA24 | ANDEAN | CONTROL | FEMALE | 60 | 0.153 | 0.669 | 0.178 |
| DSSA25 | ANDEAN | CONTROL | FEMALE | 11 | 0.154 | 0.669 | 0.177 |
| DSSA107 | ANDEAN | LL-MB | MALE | 48 | 0.584 | 0.304 | 0.113 |
| DSSA119 | ANDEAN | BL-MB | MALE | 47 | 0.278 | 0.425 | 0.296 |
| DSSA120 | ANDEAN | LL-MB | MALE | 45 | 0.193 | 0.467 | 0.34 |
| DSSA200 | ANDEAN | CONTROL | FEMALE | 59 | 0.159 | 0.584 | 0.258 |
| DSSA201 | ANDEAN | CONTROL | FEMALE | 22 | 0.137 | 0.531 | 0.332 |
| DSSA202 | ANDEAN | CONTROL | FEMALE | 39 | 0.25 | 0.518 | 0.231 |
| DSSA203 | ANDEAN | CONTROL | MALE | 13 | 0.139 | 0.728 | 0.133 |
| DSSA204 | ANDEAN | CONTROL | MALE | 81 | 0.334 | 0.462 | 0.204 |
| DSSA205 | ANDEAN | CONTROL | FEMALE | 73 | 0.159 | 0.378 | 0.464 |
| DSSA206 | ANDEAN | CONTROL | FEMALE | 75 | 0.152 | 0.524 | 0.325 |
| DSSA207 | ANDEAN | CONTROL | FEMALE | 38 | 0.157 | 0.539 | 0.304 |
| DSSA209 | ANDEAN | CONTROL | MALE | 79 | 0.22 | 0.456 | 0.324 |
| DSSA211 | ANDEAN | CONTROL | FEMALE | 12 | 0.149 | 0.386 | 0.466 |
| DSSA212 | ANDEAN | CONTROL | MALE | 23 | 0.404 | 0.417 | 0.179 |
| DSSA213 | ANDEAN | CONTROL | FEMALE | 70 | 0.149 | 0.516 | 0.335 |
| DSSA514 | ANDEAN | TT-PB | MALE | 54 | 0.204 | 0.529 | 0.267 |
| DSSA515 | ANDEAN | CONTROL | FEMALE | 44 | 0.274 | 0.457 | 0.269 |
| DSSA518 | ANDEAN | LL-MB | MALE | 74 | 0.216 | 0.479 | 0.305 |
| DSSA523 | ANDEAN | CONTROL | FEMALE | 72 | 0.181 | 0.536 | 0.283 |
| DSSA525 | ANDEAN | CONTROL | FEMALE | 72 | 0.218 | 0.554 | 0.227 |
| DSSA526 | ANDEAN | LL-MB | MALE | 48 | 0.126 | 0.678 | 0.196 |
| DSSA528 | ANDEAN | CONTROL | FEMALE | 39 | 0.273 | 0.545 | 0.182 |
| DSSA530 | ANDEAN | TT-PB | MALE | 65 | 0.219 | 0.501 | 0.28 |
| DSSA531 | ANDEAN | CONTROL | FEMALE | 56 | 0.263 | 0.536 | 0.201 |
| DSSA532 | ANDEAN | CONTROL | MALE | 25 | 0.136 | 0.673 | 0.191 |
| DSSA535 | ANDEAN | TT-PB | MALE | 40 | 0.167 | 0.63 | 0.203 |
| DSSA537 | ANDEAN | LL-MB | MALE | 50 | 0.179 | 0.571 | 0.251 |
| DSSA538 | ANDEAN | CONTROL | FEMALE | 48 | 0.186 | 0.655 | 0.158 |
| DSSA539 | ANDEAN | LL-MB | FEMALE | 57 | 0.313 | 0.51 | 0.177 |
| DSSA543 | ANDEAN | CONTROL | MALE | 13 | 0.271 | 0.498 | 0.23 |
| DSSA544 | ANDEAN | BB-PB | MALE | 76 | 0.18 | 0.539 | 0.281 |
| DSSA549 | ANDEAN | LL-MB | MALE | 44 | 0.35 | 0.413 | 0.237 |
| DSSA550 | ANDEAN | CONTROL | FEMALE | 27 | 0.272 | 0.582 | 0.146 |
| DSSA551 | ANDEAN | LL-MB | FEMALE | 49 | 0.351 | 0.507 | 0.142 |
| DSSA552 | ANDEAN | LL-MB | MALE | 45 | 0.175 | 0.525 | 0.3 |
| DSSA554 | ANDEAN | BL-MB | MALE | 29 | 0.158 | 0.419 | 0.423 |
| DSSA555 | ANDEAN | CONTROL | FEMALE | 55 | 0.109 | 0.543 | 0.348 |
| DSSA556 | ANDEAN | CONTROL | MALE | 67 | 0.126 | 0.621 | 0.253 |
| DSSA557 | ANDEAN | LL-MB | FEMALE | 59 | 0.223 | 0.626 | 0.151 |
| DSSA562 | ANDEAN | LL-MB | MALE | 64 | 0.446 | 0.389 | 0.165 |
| DSSA563 | ANDEAN | LI-PB | FEMALE | 44 | 0.251 | 0.475 | 0.275 |
| DSSA566 | ANDEAN | CONTROL | FEMALE | 18 | 0.157 | 0.476 | 0.367 |
| DSSA567 | ANDEAN | TT-PB | MALE | 51 | 0.309 | 0.516 | 0.175 |
| DSSA568 | ANDEAN | CONTROL | MALE | 50 | 0.269 | 0.521 | 0.209 |
| DSSA570 | ANDEAN | CONTROL | FEMALE | 44 | 0.365 | 0.478 | 0.157 |
| DSSA571 | ANDEAN | LL-MB | MALE | 50 | 0.149 | 0.421 | 0.43 |
| DSSA573 | ANDEAN | CONTROL | FEMALE | 46 | 0.13 | 0.67 | 0.2 |
| DSSA574 | ANDEAN | LL-MB | MALE | 74 | 0.13 | 0.53 | 0.339 |
| DSSA575 | ANDEAN | LL-MB | MALE | 33 | 0.487 | 0.347 | 0.167 |
| DSSA576 | ANDEAN | TT-PB | MALE | 52 | 0.13 | 0.604 | 0.267 |
| DSSA577 | ANDEAN | CONTROL | FEMALE | 46 | 0.103 | 0.72 | 0.177 |
| DSSA579 | ANDEAN | LL-MB | MALE | 70 | 0.142 | 0.611 | 0.247 |
| DSSA582 | ANDEAN | TT-PB | MALE | 52 | 0.124 | 0.694 | 0.182 |
| DSSA583 | ANDEAN | CONTROL | MALE | 34 | 0.144 | 0.538 | 0.318 |
| DSSA586 | ANDEAN | LL-MB | MALE | 57 | 0.15 | 0.652 | 0.199 |
| DSSA592 | ANDEAN | CONTROL | MALE | 18 | 0.251 | 0.567 | 0.182 |
| DSSA595 | ANDEAN | LL-MB | MALE | 48 | 0.56 | 0.325 | 0.116 |
| DSSA596 | ANDEAN | LL-MB | MALE | 41 | 0.236 | 0.56 | 0.204 |
| DSSA601 | ANDEAN | LL-MB | MALE | 57 | 0.378 | 0.482 | 0.141 |
| DSSA604 | ANDEAN | CONTROL | FEMALE | 54 | 0.34 | 0.516 | 0.144 |
| DSSA605 | ANDEAN | CONTROL | FEMALE | 9 | 0.286 | 0.564 | 0.15 |
| DSSA606 | ANDEAN | CONTROL | FEMALE | 9 | 0.234 | 0.579 | 0.187 |
| DSSA608 | ANDEAN | CONTROL | FEMALE | 28 | 0.151 | 0.404 | 0.445 |
| DSSA609 | ANDEAN | LL-MB | MALE | 5 | 0.261 | 0.305 | 0.434 |
| DSSA610 | ANDEAN | CONTROL | MALE | 56 | 0.333 | 0.425 | 0.243 |
| DSSA611 | ANDEAN | TT-PB | MALE | 41 | 0.234 | 0.4 | 0.366 |
| DSSA615 | ANDEAN | BL-MB | MALE | 60 | 0.238 | 0.567 | 0.195 |
| DSSA616 | ANDEAN | BL-MB | MALE | 64 | 0.254 | 0.618 | 0.128 |
| DSSA617 | ANDEAN | CONTROL | FEMALE | 51 | 0.152 | 0.586 | 0.261 |
| DSSA650 | ANDEAN | LL-MB | MALE | 26 | 0.452 | 0.405 | 0.142 |
| DSSA657 | ANDEAN | LL-MB | MALE | 55 | 0.308 | 0.475 | 0.216 |
| DSSA661 | ANDEAN | LL-MB | FEMALE | 74 | 0.507 | 0.344 | 0.149 |
| DSSA662 | ANDEAN | LI-PB | MALE | 61 | 0.356 | 0.377 | 0.266 |
| DSSA664 | ANDEAN | LL-MB | MALE | 51 | 0.294 | 0.525 | 0.181 |
| DSSA665 | ANDEAN | CONTROL | FEMALE | 52 | 0.321 | 0.379 | 0.3 |
| DSSA669 | ANDEAN | LL-MB | MALE | 66 | 0.28 | 0.552 | 0.168 |
| OPS3 | ATLANTIC | CONTROL | FEMALE | 58 | 0.259 | 0.516 | 0.225 |
| OPS4 | ATLANTIC | LL-MB | MALE | 75 | 0.555 | 0.325 | 0.12 |
| OPS5 | ATLANTIC | CONTROL | FEMALE | 52 | 0.301 | 0.494 | 0.205 |
| OPS6 | ATLANTIC | CONTROL | MALE | 15 | 0.163 | 0.622 | 0.215 |
| OPS7 | ATLANTIC | CONTROL | MALE | 5 | 0.26 | 0.592 | 0.148 |
| OPS8 | ATLANTIC | LL-MB | MALE | 73 | 0.298 | 0.494 | 0.208 |
| OPS9 | ATLANTIC | BL-MB | FEMALE | 78 | 0.109 | 0.425 | 0.466 |
| OPS10 | ATLANTIC | LL-MB | MALE | 72 | 0.169 | 0.594 | 0.237 |
| OPS13 | ATLANTIC | LL-MB | MALE | 54 | 0.175 | 0.574 | 0.251 |
| OPS15 | ATLANTIC | CONTROL | FEMALE | 54 | 0.131 | 0.672 | 0.197 |
| OPS18 | ATLANTIC | CONTROL | MALE | 25 | 0.139 | 0.446 | 0.415 |
| OPS25 | ATLANTIC | TT-PB | MALE | 72 | 0.285 | 0.452 | 0.263 |
| OPS36 | ATLANTIC | LL-MB | FEMALE | 44 | 0.159 | 0.448 | 0.393 |
| OPS42 | ATLANTIC | CONTROL | FEMALE | 29 | 0.307 | 0.557 | 0.136 |
| OPS45 | ATLANTIC | LL-MB | MALE | 61 | 0.277 | 0.476 | 0.246 |
| OPS47 | ATLANTIC | CONTROL | FEMALE | 27 | 0.306 | 0.529 | 0.165 |
| OPS53 | ATLANTIC | LL-MB | MALE | 16 | 0.307 | 0.467 | 0.227 |
| OPS59 | ATLANTIC | BL-MB | FEMALE | 85 | 0.239 | 0.597 | 0.163 |
| OPS60 | ATLANTIC | CONTROL | FEMALE | 50 | 0.376 | 0.513 | 0.111 |
| OPS61 | ATLANTIC | CONTROL | FEMALE | 8 | 0.311 | 0.565 | 0.124 |
| OPS62 | ATLANTIC | LL-MB | FEMALE | 20 | 0.351 | 0.542 | 0.107 |
| OPS63 | ATLANTIC | LL-MB | FEMALE | 22 | 0.342 | 0.517 | 0.14 |
| OPS64 | ATLANTIC | CONTROL | FEMALE | 28 | 0.465 | 0.418 | 0.117 |
| OPS65 | ATLANTIC | LL-MB | FEMALE | 50 | 0.159 | 0.505 | 0.336 |
| OPS66 | ATLANTIC | CONTROL | MALE | 52 | 0.264 | 0.464 | 0.272 |
| OPS72 | ATLANTIC | CONTROL | MALE | 39 | 0.285 | 0.484 | 0.231 |
| OPS74 | ATLANTIC | LL-MB | FEMALE | 44 | 0.312 | 0.506 | 0.183 |
| OPS75 | ATLANTIC | LL-MB | MALE | 75 | 0.366 | 0.49 | 0.144 |
| OPS76 | ATLANTIC | CONTROL | FEMALE | 74 | 0.179 | 0.647 | 0.173 |
| OPS77 | ATLANTIC | CONTROL | MALE | 40 | 0.325 | 0.383 | 0.291 |
| OPS83 | ATLANTIC | CONTROL | MALE | 50 | 0.315 | 0.44 | 0.245 |
| OPS84 | ATLANTIC | CONTROL | MALE | 15 | 0.288 | 0.465 | 0.248 |
| OPS92 | ATLANTIC | LL-MB | MALE | 71 | 0.336 | 0.49 | 0.174 |
| OPS94 | ATLANTIC | CONTROL | FEMALE | 13 | 0.321 | 0.465 | 0.214 |
| OPS95 | ATLANTIC | LL-MB | MALE | 44 | 0.29 | 0.404 | 0.306 |
| OPS96 | ATLANTIC | CONTROL | FEMALE | 35 | 0.421 | 0.377 | 0.202 |
| OPS97 | ATLANTIC | LL-MB | MALE | 52 | 0.29 | 0.451 | 0.259 |
| OPS98 | ATLANTIC | CONTROL | FEMALE | 51 | 0.299 | 0.509 | 0.192 |
| OPS102 | ATLANTIC | LL-MB | MALE | 46 | 0.445 | 0.434 | 0.121 |
| OPS105 | ATLANTIC | LL-MB | MALE | 59 | 0.433 | 0.389 | 0.178 |
| OPS107 | ATLANTIC | CONTROL | FEMALE | 31 | 0.321 | 0.398 | 0.282 |
| OPS109 | ATLANTIC | BL-MB | FEMALE | 31 | 0.378 | 0.463 | 0.158 |
| OPS110 | ATLANTIC | BL-MB | MALE | 9 | 0.335 | 0.501 | 0.164 |
| OPS112 | ATLANTIC | CONTROL | MALE | 33 | 0.417 | 0.372 | 0.21 |
| OPS116 | ATLANTIC | CONTROL | MALE | 19 | 0.34 | 0.461 | 0.199 |
| OPS121 | ATLANTIC | CONTROL | FEMALE | 32 | 0.398 | 0.426 | 0.176 |
| OPS122 | ATLANTIC | LL-MB | MALE | 29 | 0.515 | 0.371 | 0.113 |
| OPS124 | ATLANTIC | BL-MB | FEMALE | 44 | 0.47 | 0.406 | 0.124 |
| OPS127 | ATLANTIC | LL-MB | MALE | 32 | 0.428 | 0.415 | 0.157 |
| OPS130 | ATLANTIC | CONTROL | FEMALE | 27 | 0.558 | 0.331 | 0.111 |
| OPS134 | ATLANTIC | CONTROL | MALE | 18 | 0.219 | 0.544 | 0.237 |
| OPS135 | ATLANTIC | CONTROL | MALE | 22 | 0.192 | 0.483 | 0.326 |
| OPS136 | ATLANTIC | BL-MB | MALE | 70 | 0.375 | 0.492 | 0.133 |
| OPS137 | ATLANTIC | CONTROL | FEMALE | 59 | 0.521 | 0.367 | 0.111 |
| OPS142 | ATLANTIC | LL-MB | MALE | 51 | 0.328 | 0.364 | 0.308 |
| OPS144 | ATLANTIC | CONTROL | FEMALE | 29 | 0.443 | 0.415 | 0.143 |
| OPS204 | ATLANTIC | LL-MB | MALE | 74 | 0.355 | 0.445 | 0.2 |
| OPS205 | ATLANTIC | CONTROL | FEMALE | 70 | 0.457 | 0.363 | 0.18 |
| OPS207 | ATLANTIC | CONTROL | MALE | 18 | 0.288 | 0.485 | 0.227 |
| OPS209 | ATLANTIC | LL-MB | MALE | 70 | 0.353 | 0.473 | 0.174 |
| OPS210 | ATLANTIC | CONTROL | FEMALE | 53 | 0.327 | 0.528 | 0.145 |
| OPS214 | ATLANTIC | LL-MB | MALE | 29 | 0.648 | 0.275 | 0.077 |
| OPS217 | ATLANTIC | CONTROL | FEMALE | 17 | 0.423 | 0.373 | 0.204 |
| OPS224 | ATLANTIC | CONTROL | FEMALE | 26 | 0.343 | 0.519 | 0.139 |
| OPS225 | ATLANTIC | CONTROL | MALE | 28 | 0.286 | 0.396 | 0.317 |
| OPS226 | ATLANTIC | LL-MB | MALE | 50 | 0.285 | 0.545 | 0.169 |
| OPS227 | ATLANTIC | LL-MB | MALE | 54 | 0.17 | 0.558 | 0.272 |
| OPS228 | ATLANTIC | CONTROL | FEMALE | 15 | 0.182 | 0.498 | 0.32 |
| OPS234 | ATLANTIC | CONTROL | MALE | 18 | 0.302 | 0.551 | 0.147 |
| OPS239 | ATLANTIC | CONTROL | MALE | 9 | 0.215 | 0.581 | 0.204 |
| OPS240 | ATLANTIC | LL-MB | FEMALE | 67 | 0.451 | 0.427 | 0.122 |
| OPS241 | ATLANTIC | CONTROL | FEMALE | 17 | 0.49 | 0.381 | 0.129 |
| OPS242 | ATLANTIC | CONTROL | FEMALE | 22 | 0.379 | 0.497 | 0.123 |
| OPS244 | ATLANTIC | CONTROL | MALE | 2 | 0.429 | 0.435 | 0.135 |
| OPS245 | ATLANTIC | CONTROL | FEMALE | 3 | 0.373 | 0.445 | 0.181 |
| OPS246 | ATLANTIC | CONTROL | FEMALE | 9 | 0.546 | 0.354 | 0.1 |
| OPS247 | ATLANTIC | CONTROL | FEMALE | 6 | 0.415 | 0.417 | 0.168 |
| OPS248 | ATLANTIC | CONTROL | MALE | 30 | 0.25 | 0.53 | 0.221 |

*LL= lepromatous leprosy, BL: borderline lepromatous leprosy, BB: borderline borderline leprosy, BT: borderline tuberculoid leprosy, TT: tuberculoid leprosy. MB=multibacillary leprosy. PB=paucibacillary leprosy. ** INDIVIDUAL ANCESTRAL COMPOSITION= African+European+Native-American= 1.
