# Supplementary figures and images for: Human Genetic Ancestral Composition Correlates with the Origin of Mycobacterium leprae Strains in a Leprosy Endemic Population
Source: PLoS Negl Trop Dis. 2015 Sep 11;9(9):e0004045. doi: 10.1371/journal.pntd.0004045 (PMC4567314; doi:10.1371/journal.pntd.0004045)

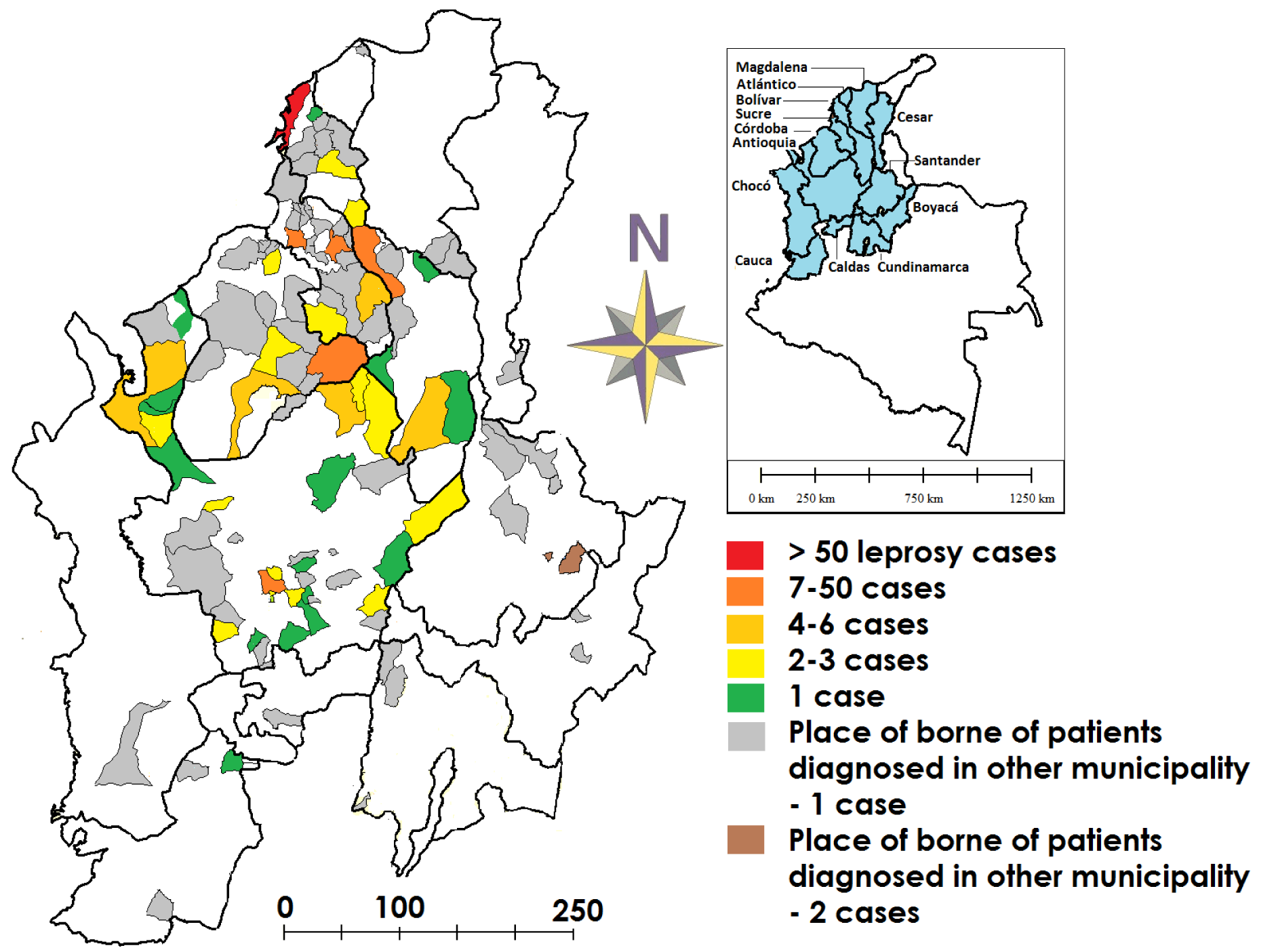

Supplement: S1 Fig — (TIF) [file pntd.0004045.s003.tif]
